# Investigating the Effects of Hyperbaric Oxygen Treatment in Necrotizing Soft Tissue Infection With Transcriptomics and Machine Learning (the HBOmic Study): Protocol for a Prospective Cohort Study With Data Validation

**DOI:** 10.2196/39252

**Published:** 2022-11-25

**Authors:** Julie Vinkel, Leonor Rib, Alfonso Buil, Morten Hedetoft, Ole Hyldegaard

**Affiliations:** 1 Department of Anaesthesiology Copenhagen University Hospital, Rigshospitalet Copenhagen Denmark; 2 Department of Clinical Medicine University of Copenhagen Copenhagen Denmark; 3 Biotech Research and Innovation Centre University of Copenhagen Copenhagen Denmark; 4 Institute for Biological Psychiatry Center of Psychiatry Sankt Hans Roskilde Denmark; 5 Department of Anaesthesiology Zealand University Hospital Køge Denmark

**Keywords:** necrotizing soft tissue infection, hyperbaric oxygen treatment, host-pathogen interaction, transcriptomic, sepsis, machine learning, infection, soft tissue infection, study protocol, NSTI, treatment, oxygen, tissue, data, validation, immunology, immune system, mechanism, response, genome, longitudinal, biomarker, signaling

## Abstract

**Background:**

Necrotizing soft tissue infections (NSTIs) are complex multifactorial diseases characterized by rapid bacterial proliferation and progressive tissue death. Treatment is multidisciplinary, including surgery, broad-spectrum antibiotics, and intensive care; adjunctive treatment with hyperbaric oxygen (HBO_2_) may also be applied. Recent advances in molecular technology and biological computation have given rise to new approaches to infectious diseases based on identifying target groups defined by activated pathophysiological mechanisms.

**Objective:**

We aim to capture NSTI disease signatures and mechanisms and responses to treatment in patients that receive the highest standard of care; therefore, we set out to investigate genome-wide transcriptional responses to HBO_2_ treatment during NSTI in the host and bacteria.

**Methods:**

The Effects of Hyperbaric Oxygen Treatment Studied with Omics (HBOmic) study is a prospective cohort study including 95 patients admitted for NSTI at the intensive care unit of Copenhagen University Hospital (Rigshospitalet), Denmark, between January 2013 and June 2017. All participants were treated according to a local protocol for management of NSTI, and biological samples were obtained and stored according to a standard operational procedure. In the proposed study, we will generate genome-wide expression profiles of whole-blood samples and samples of infected tissue taken before and after HBO_2_ treatment administered during the initial acute phase of infection, and we will analyze the profiles with unsupervised hierarchical clustering and machine learning. Differential gene expression will be compared in samples taken before and after HBO_2_ treatment (N=85), and integration of profiles from blood and tissue samples will be performed. Furthermore, findings will be compared to NSTI patients who did not receive HBO_2_ treatment (N=10). Transcriptomic data will be integrated with clinical data to investigate associations and predictors.

**Results:**

The first participant was enrolled on July 27, 2021, and data analysis is expected to begin during autumn 2022, with publication of results immediately thereafter.

**Conclusions:**

The HBOmic study will provide new insights into personalized patient management in NSTIs.

**Trial Registration:**

ClinicalTrials.gov NCT01790698; https://clinicaltrials.gov/ct2/show/NCT01790698

**International Registered Report Identifier (IRRID):**

DERR1-10.2196/39252

## Introduction

Necrotizing soft tissue infections (NSTIs) are severe infections of the soft tissue surrounding the bones that are frequently accompanied by septic shock and multiorgan failure. Advanced supportive care in the intensive care unit is frequently required [1]. The reported mortality rate of NSTIs varies among studies and countries, with an overall mortality of 24% and average 30-day mortality rates of 20% to 40% [2-5]. In our cohort, we found a 30-day mortality rate of 14%, and amputation was performed in up to 13% of cases [6]. The infections are characterized by rapid bacterial proliferation and progressive tissue destruction of the fascia and deep skin layers. This process is triggered by white blood cell infiltration causing thrombosis of the veins and arteries perforating the fascia. Accompanied by further microorganism proliferation and biofilm formation, this progresses to the occlusion of nutrient vessels with subsequent ischemia and tissue death and potentially reduced antibiotic effects [7-9].

The contemporary treatment strategy is a combination of empirical broad-spectrum antimicrobial therapy, aggressive surgical debridement, and cardiovascular support. Hyperbaric oxygen (HBO_2_) treatment is used worldwide as an adjunctive treatment in NSTI as a means of reducing tissue loss and death [10-13]. Treatment with HBO_2_ leads to hyperoxia. Besides improving oxygen supply to hypoxic and ischemic tissues, it has been suggested that HBO_2_ treatment promotes beneficial immunomodulatory activities and antibacterial actions [14-17], resulting in improved survival [18-20]. The immunomodulatory effects of HBO_2_ treatment for infectious diseases include coagulopathy, endothelial activation, and altered cellular metabolism; these effects have been revealed using the traditional approach of biomarker discovery [21-25]. However, the mechanisms of action of HBO_2_ treatment for NSTI on a molecular gene-expression level and the genetic associations with clinical and demographic variables have not yet been investigated, and a coherent understanding of the pathophysiological effects of HBO_2_ in NSTIs is needed [10].

NSTIs are caused by a variety of microbes, and affected patients are highly heterogeneous, including both young immunocompetent individuals and individuals with severe comorbidities [5]. The disease complex may be driven as much by the biology of the host response as by the type of microbe invading the host. This view is supported by earlier studies that show that NSTI patients infected with group A *Streptococcus* were more likely to develop septic shock [6]. It is not surprising that the human genome holds variants related to infection, given that infectious diseases have been the largest cause of death during our evolution [26]. The multifaceted, heterogeneous nature of disease may explain the lack of success with identification of biomarkers. Bacterial toxin–mediated inflammation is associated with altered expression of more than 3700 human genes, making gene-expression analysis a potentially useful tool for discovery-oriented studies of the pathogenesis of sepsis and severe infections [27]. Traditional analysis of exposure and outcome does not fully utilize the power of combined gene expression data [28]. This paper presents a transcriptomic study protocol for examining host and pathogen interactions using a data-driven approach with unsupervised analysis. Our hypotheses are that genetic diversity accounts for the variation in outcomes that follow interactions between humans and the potentially life-threatening pathogens in NSTIs and that these interactions are modulated by HBO_2_ treatment.

## Methods

### Study Design and Setting

**Figure 1:**
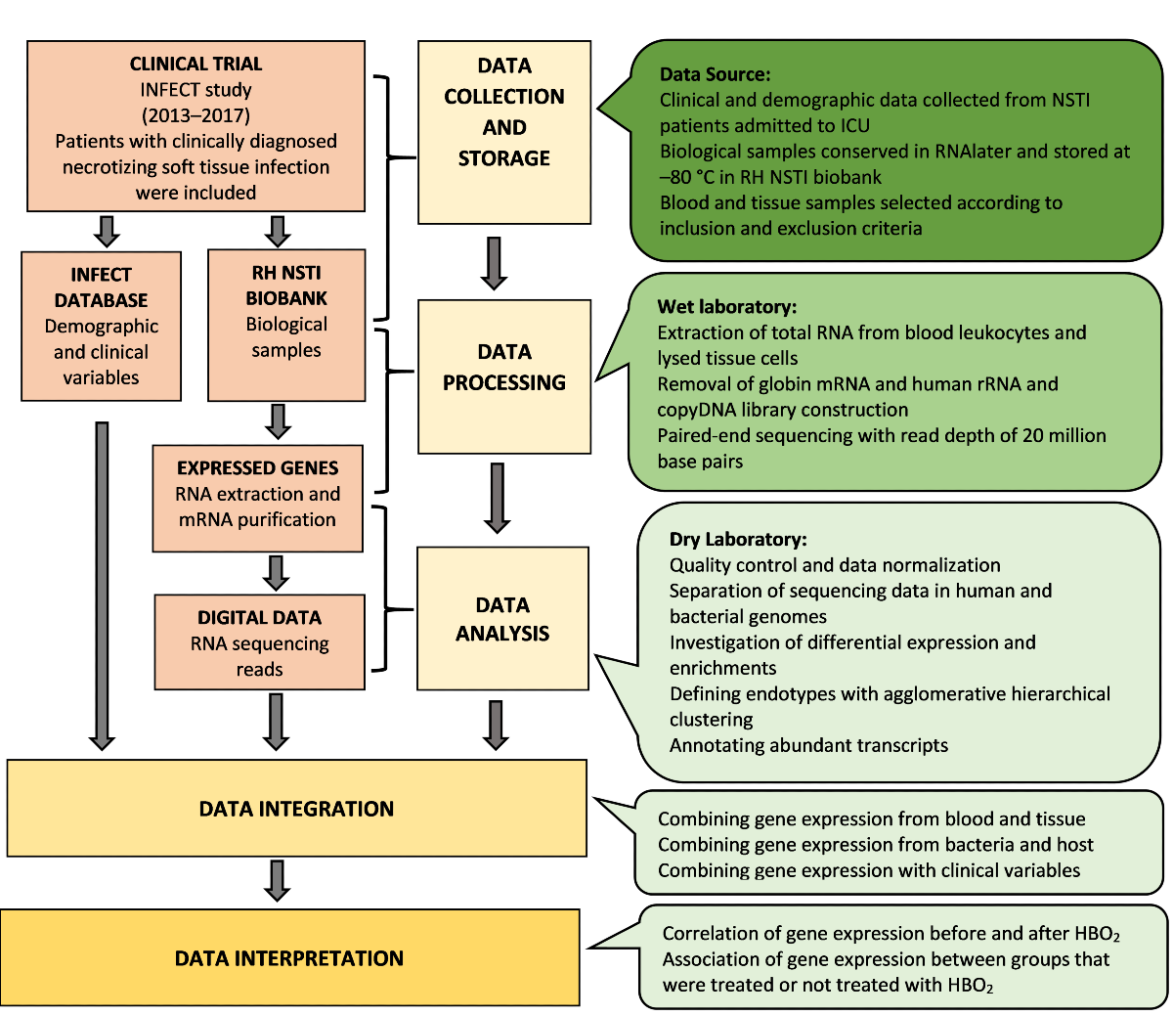
Workflow of the HBOmic study. HBO_2_: hyperbaric oxygen treatment; ICU: intensive care unit; INFECT: Systems Medicine to Study Necrotizing Soft Tissue Infections; mRNA: messenger RNA; NSTI: necrotizing soft tissue infection; RH: Rigshospitalet.

This is a prospective observational study. All participants were enrolled in the Systems Medicine to Study Necrotizing Soft Tissue Infections (INFECT) study, a clinical study that systematically collected blood and tissue samples with the purpose of including these samples in a biobank for bioinformatics studies of large biochemical groups. For the Effects of Hyperbaric Oxygen Treatment Studied with Omics (HBOmic) study, we will screen this biobank (the Rigshospitalet NSTI biobank) for patients that meet our eligibility criteria, starting with the 2017 data and working backward until we meet our desired sample size. The HBOmic study will analyze gene expression in samples of peripheral blood leukocytes and samples from infected tissue sites. The transcriptome will be compared in patients before and after they undergo HBO_2_ treatment and in patients who were and were not treated with HBO_2_. The workflow of the study is illustrated in Figure 1.

### Eligibility Criteria

Participants are included in the HBO_2_ treatment group if they had been clinically diagnosed with NSTI according to previously defined criteria [6], had been treated with HBO_2_ during the initial acute phase of the infection, had blood samples withdrawn before and after HBO_2_ treatment, and had been subjected to surgical debridement with sampling of infected tissue before and after HBO_2_ treatment. Participants are included in the non–HBO_2_ treatment group if they had been clinically diagnosed with NSTI according to previously defined criteria [6], had 2 blood samples withdrawn at different time points during the initial acute phase of the infection, had been subjected to surgical debridement twice with sampling of infected tissue at 2 different time points during the initial acute phase of the infection, and had not been treated with HBO_2_ between sample collection. Participants were excluded from the study if they were alive and unwilling or unable to give informed consent.

### HBO_2_ Intervention

The HBO_2_ treatment was performed in a hyperbaric multichamber (Drass Galeazzi SpA, Type HPO4000, HPE50.2.A) that had been modified to deliver intensive care treatment during pressurization, including mechanical ventilation (Servo-I-30 HBO Editor, Maquet), cardiovascular monitoring (Intellivue, Phillips, MP30), and multiple intravascular infusions (Perfusor Space, Braun). All participants who underwent HBO_2_ treatment were treated according a standardized treatment protocol, which aimed at a minimum of 3 HBO_2_ treatments, with the first treatment administered as soon as possible after hospital admission. The treatment duration of each session was 90 minutes at a pressure of 284 kPa without air breaks and a compression and decompression rate of 15 minutes.

Concomitant care was also protocolized and aimed at 3 surgical revisions during the first 24 hours after diagnosis, with repeated revisions thereafter as necessary. Antibiotic treatment with meropenem, ciprofloxacin, and clindamycin and intensive care treatment were adapted to individual needs [6].

### Studies and Outcomes

Study 1 aims to obtain transcriptome profiles of peripheral whole blood before and after HBO_2_ treatment to reveal treatment-dependent gene regulation of the septic response to infection. The primary outcome will be the change in gene expression in whole-blood samples before and after HBO_2_ treatment. In study 2, we will perform simultaneous transcriptional profiling of infected human tissue and bacterial gene expression before and after HBO_2_ treatment to reveal treatment-dependent alterations in microbial virulence mechanisms and interactions with the host immune system. The primary outcome will be the change in gene expression in samples of infected tissue before and after HBO_2_ treatment. Study 3 aims to integrate the molecular response to sepsis with the immune response that unfolds in the tissues that are the source of the NSTI, including changes in the response to treatment with HBO_2_ treatment. The primary outcome will be the correlation of the transcriptional profile of the whole blood and infected human tissue in patients with NSTI, before and after HBO_2_ treatment.

### Study Population

All participants were diagnosed with NSTI by the surgeon at the primary operation. The diagnosis was based on the presence of necrotic or deliquescent soft tissue with widespread undermining of the surrounding tissue, as determined by the surgeon. The diagnosis was systematically cross-checked by study investigators, and patients were excluded from the study if necrotic or deliquescent tissue was not described in the patient records. Detailed characteristics of the participant population have been published elsewhere [6].

### Participant Timeline

**Figure 2:**
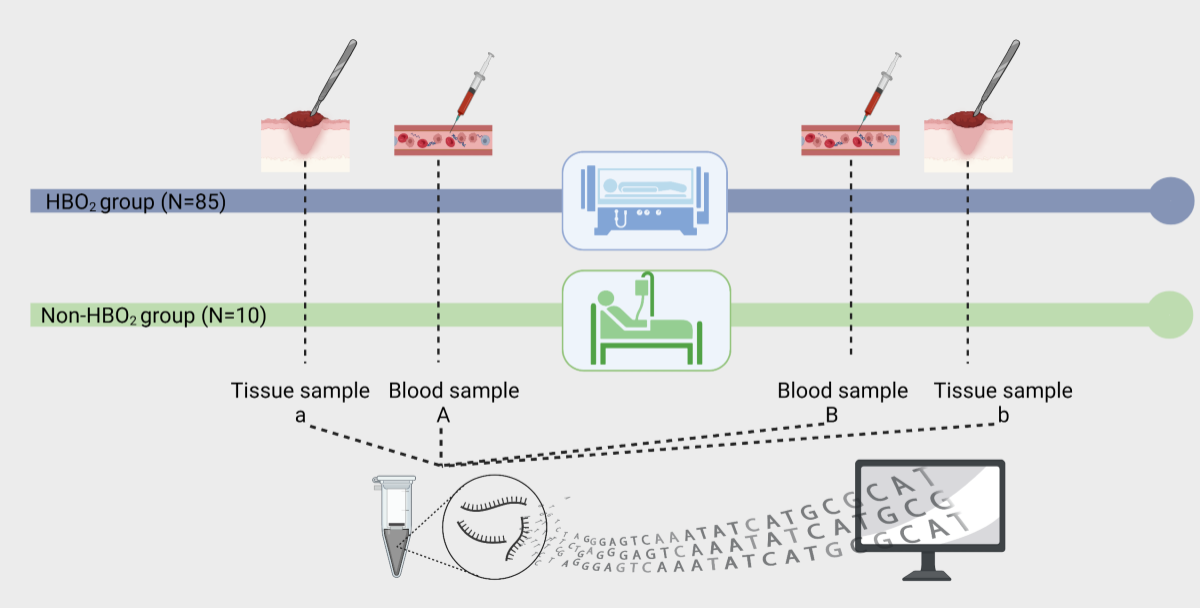
Participant Timeline. Whole-blood samples and tissue biopsies taken during surgical debridement were stored in RNAlater. All samples were immediately frozen and stored at –80 °C until processing. Hyperbaric oxygen treatment was administered at 284 kPa for a minimum of 90 minutes. Patients had received 0 (ie, the non–hyperbaric oxygen group), 1, or 2 hyperbaric oxygen sessions when the follow-up samples “B” and “b” were taken.

Participants included in the study followed the participant timeline illustrated in Figure 2.

### Data Collection and Storage

#### Clinical Variables

All clinical variables for the included participants are available through the INFECT project’s INFECT database, which contains more than 2000 variables, including baseline variables, time variables related to hospital admission, variables monitored at the intensive care unit and during surgical procedures, tissue sampling and microbiological findings, and variables related to long-term outcomes and quality of life measures. A full list of variables is available in the appendix to a previous publication [29].

#### Biological Samples

Each tissue biopsy was collected during surgical debriding. Sample volumes ranged from 0.5 to 0.75 cm^2^. Immediately after collection, the tissue was placed in a 1-ml sterile natural tube (Cryo.s) and covered and stabilized in 0.5 ml RNAlater (Thermo Fisher Scientific). A tissue specimen of the same size was also placed in a 1-ml sterile natural tube (Cryo.s) without RNAlater. Then, the tubes were placed directly in a thermal container on dry ice and transported to a freezer in the same building, where they were frozen at –80 °C for storage. All tissue samples were previously categorized by type of tissue (muscle, fascia, or soft tissue), the degree to which the tissue was affected by infection (normal, infected without necrosis, or necrotic) and whether it was collected from the margin or the center of the infection. This categorization of the tissue samples was a subjective clinical categorization performed by the surgeon in the acute setting based on the look and texture of the biopsied area, not a pathological, microscopically verified classification. We selected samples in accord with a predefined strategy; the categorization of each sample was noted.

Whole blood was collected with an arterial catheter using a 10-ml lithium heparin tube; 2.5 ml of the blood was transferred to a sterile natural tube (Cellstar) with a sterile 10-ml syringe and mixed with 5 ml of RNAlater. The content was mixed by turning the tubes upside down a few times before they were transported on dry ice to a freezer in the same building, where they were stored at –80 °C. All samples were handled with sterile procedures. Blood was collected with the vacuum technique, and a discard tube was used prior to blood collection.

The blood and tissue samples included in this study were stabilized with RNAlater (Thermo Fisher Scientific). The data and time of sampling were noted, along with any deviations from standard operational procedures.

### Ethics Approval

The study presented in this protocol abides by the principles outlined in the Declaration of Helsinki. The INFECT study is registered at ClinicalTrials.gov (NCT01790698). During the INFECT study, informed consent for collection and storage of the biological material for future research was given. We will follow the genomics guidelines of the Danish National Committee on Health Research Ethics, including the special requirements for research projects involving extensive mapping. Also following the Danish National Committee on Health Research Ethics (journal number 2010299, locally in journal number 1151739), renewed informed consent for this study was obtained from living participants. This study was approved by Capital Region at Knowledge Center for Data Reviews (P-2020-1186).

### Data Processing

#### RNA Purification, Library Preparation, and Sequencing

A volume of 300 to 500 μL of anticoagulated whole blood will be used to extract total RNA from leukocytes using the RiboPure RNA Purification Kit (Thermo Fisher Scientific). Infected tissue biopsies (10-30 mg) will be disrupted and homogenized with TissueLyser (Qiagen), and total RNA of both human and bacterial origin will be isolated using the RNeasy Plus Mini Kit (Qiagen). We will use the NEBNext Globin & rRNA Depletion Kit (New England Biolabs Inc) for strand-specific messenger RNA (mRNA) purification using probes that are selective for globin mRNA, cytoplasmic ribosomal RNA (rRNA), and mitochondrial rRNA with human, mouse, and rat samples. In all biological samples, first- and second-strand copyDNA (cDNA) will be synthesized based on ligation adaptor techniques (NEBNext Globin & rRNA Depletion Kit [human, mouse, and rat]). Each step will be performed according to the manufacturer’s instructions and an internal standard operational procedure. The obtained cDNA library will then be sequenced with dual RNA sequencing with paired-end sequencing of 150 nucleotide fragments on the Illumina Novaseq6000 platform with a targeted sequencing read depth of 20 million reads.

### Data Validation and Quality Check

We have performed a validation test of the data processing method using 8 tissue samples and 8 whole blood samples to estimate the quantity and integrity of the extracted RNA, the quality of the cDNA libraries, and the sequencing output.

#### Quality Check Methods

All data processing was performed as described in this protocol. The quality check of the extracted RNA and the prepared libraries was performed on a Fragment Analyzer with the included ProSize software (version 3.0; Agilent Technologies, Inc). Quantity was measured with Qubit fluorometric quantification. Quality checks of the RNA sequence data were performed in MultiQC (version 1.7; Seqera Labs). We performed computational calculation of the transcript integrity number (TIN) as a measure of the RNA degradation level across all the transcripts that were annotated in the computational databases [30].

#### Quality Check Results

The results of the pilot quality check are summarized in Table 1.

**Table 1:** Results of the quality check.

Measurements	Whole blood samples	Infected tissue samples
RNA quantity (ng), median (IQR)	6560 (2540)	830 (2471)
RNA quality number, mean (SD)	9.6 (0.37)	2.9 (1.55)
Illumina sequencing Q30 quality score^a^, median (IQR)	93.55 (0.4425)	93.49 (0.245)
Obtained reads per sample (n), median (IQR)	29,178,943 (8,718,838)	31,009,818 (5,969,336)
Transcript integrity number, mean (SD)	57.7 (1.92)	55.0 (5.83)
Coefficient of variation, mean (SD)	0.34 (0.346)	0.82 (0.73)

^a^Indicates the probability of 1 in 1000 incorrect base calls.

In 1 tissue sample, we were not able to extract any RNA; this sample was thus excluded from further analysis. In the remaining 15 samples, we obtained sufficient RNA in both the whole-blood and tissue samples. The RNA in the whole-blood samples was of high quality (the RNA quality number [RQN] ranged from 8.8 to 10.0), but the RQN in the tissue samples was low. Transcript quality control and a preliminary bioinformatics analysis showed acceptable values, with average TINs of 58 (SD 1.92) and 55 (SD 5.83) for the whole-blood and tissue samples, respectively, along with a good quality of reads and a high number of unique reads.

Using the number of reads per gene, the weighted trimmed mean of the M (log ratio) values, and the TIN, we defined a linear model to calculate coefficients of variation of 0.34 (SD 0.346) and 0.82 (SD 0.73) for the whole-blood and infected tissue samples, respectively, with the estimateDisp method in the Bioconductor package edgeR. Descriptive statistics were calculated in R (version 4.1.2).

#### Evaluation of Quality Check

The infected tissue samples were collected from soft tissue with varying degrees of necrosis, and therefore varying degrees of degradation were already expected in vivo. Necrosis is premature cell death, and although it is believed that necrosis causes RNA degradation in its later stages, the order of progress of RNA decay is unknown [31]. Based on a visual inspection of electropherograms, the RNA in some of the tissue samples appeared more degraded than the whole-blood samples, as also reflected in the lower RQN. However, tissue samples are also generally expected to have higher prokaryotic RNA content than blood samples; therefore, the calculated RQN in these infected tissue samples might have been falsely low. The ProSize software can only be programmed to analyze either eukaryotic or prokaryotic rRNA. When calculating the RQN, ProSize considers the entire electropherogram, including the small and large ribosomal peaks, the baseline resolution between them, and the degradation in front of the small ribosomal peak. As the prokaryotic ribosomal RNA complexes are smaller than the eukaryotic ribosomal complexes, they will appear in front of the eukaryotic complexes, resulting in a falsely low RQN value. Moreover, the tissue samples used in this study are degraded in vivo and high levels of degradation are known to affect the reliability of the RQN [32].

Based on the findings in the quality check, we decided to adhere to the strategy for data processing presented in the protocol. We will not dismiss samples due to a low RQN calculation alone. Biological samples with ≥100 ng of RNA may proceed to library preparation irrespective of the calculated RQN value, depending on a visual inspection of the electropherogram. The TIN will be computed after mapping the sequenced reads onto the human genome to obtain an estimate of human RNA degradation on the transcript level. Libraries with sufficient RNA and an inset fragment size of >300 base pairs will continue to next-generation sequencing.

### Data Analysis

#### Quality Assessment of the Sequencing Data

Quality control of sequence reads will be done using the tools FastQC (version 0.11.2), RSeQC (version 2.6.4) [33], and fastq_screen (version 0.11.4). The proportion of human rRNA reads will be checked with the split_bam.py tool in RSeQC. Quality control will be performed separately for reads of human and bacterial origin. The cutoff for the percentage of duplicate reads and the number of unique reads will be defined post hoc to minimize the number of excluded samples while considering the risk of introducing biases to the analysis. The median TIN will be calculated for all samples to evaluate RNA degradation on the transcript level. The influence of RNA degradation on the results will be evaluated by performing a sensitivity analysis.

#### Alignment of Reads to the Genomes of Interest

Sequencing reads will be aligned onto the human and bacterial genomes. Adaptors, low-quality bases, the first 12 bases, and reads shorter than 25 nucleotides will be removed with Trimmomatic [34].

Reads will be mapped using STAR software (version 2.7.3a) separately against the human and bacterial genomes [35]. Up to 2 mismatches will be allowed during mapping, and the minimum number of overlap bases to trigger mate merging and realignment will be set to 5. Otherwise, default settings will be used. Duplicate reads will be removed using the MarkDuplicates function of Picard software.

The featureCounts function of the Rsubread R package will be used to quantify reads in exons. Bacterial typing will be performed by assigning species- and genus-level annotations to a phylotype of previously described isolates using relevant databases to identify microbiomes [36].

#### Clustering Analyses

Interindividual heterogeneity will be investigated using unsupervised hierarchical clustering for the most variable probes. Endotypes will be defined by agglomerative hierarchical clustering from samples taken before HBO_2_ therapy according to the microbial community composition, the microbial expressed virulence mechanisms, and the host immune response. Clusters will be tested for quality and stability by multiple iterations, and group membership will be consolidated using *k* means to ensure that membership is due to true cluster structure rather than stochastic picks.

#### Differential Expression Assessment

Differential gene expression analysis will be performed using edgeR or similar software by comparing individual patients and patients stratified to each of the endotypes and by comparing each endotype to the other endotypes [37]. The data will be fit to a gene-wise generalized linear model that will include relevant covariates, such as the degradation level of the samples (ie, the TIN). Differential gene usage will be assessed by quasi-likelihood tests and adjusted for multiple comparisons with the false discovery rate. Gene coexpression networks will be determined to obtain insight into the biological function of the included genes.

#### Functional Enrichment of Gene Sets

Genes showing treatment-dependent differential regulation will be explored, as will genes involved in gene networks and signaling pathways related to, for example, host immunity, inflammation, and redox homeostasis. Gene set enrichment analyses will be performed with ranked log fold changes using state-of-the-art enrichment tools, such as the gseGO function in the clusterProfiler R package [38]. Related functions will be used to visualize the results together with state-of-the-art enrichment tools, such as the DOSE R package functions [39].

### Data Integration and Interpretation

Differential gene expression data discovered before and after treatment with HBO_2_ will be compared with gene expression profiles from blood and tissue samples taken at similar time points from NSTI patients that were not treated with HBO_2_. Participants from the HBO_2_ group will be matched with participants from the non-HBO_2_ group on key variables in the downstream gene expression analyses.

Furthermore, starting from the endotypes identified before HBO_2_ treatment, we will annotate endotypes and monitor immunomodulatory effects of HBO_2_ treatment as dynamic changes in the endotypes.

Highly abundant microbial genera expressed in the initial clusters will be compared to relevant virulence factor databases before and after HBO_2_ treatment. The microbial diversity associated with NSTIs and how their pathological mechanisms may be altered in response to HBO_2_ treatment will be integrated with the host immune response.

A comparison between whole blood and infected tissue will be performed with a quantitative and qualitative comparison of the clusters obtained from the 2 tissue types before and after HBO_2_ treatment. Endotypes or subgroups will be associated with demographic and clinical variables in the INFECT database. Linear models will be applied to fit group membership with gene expression and clinical variables and identify predictors.

### Sample Size

**Figure 3:**
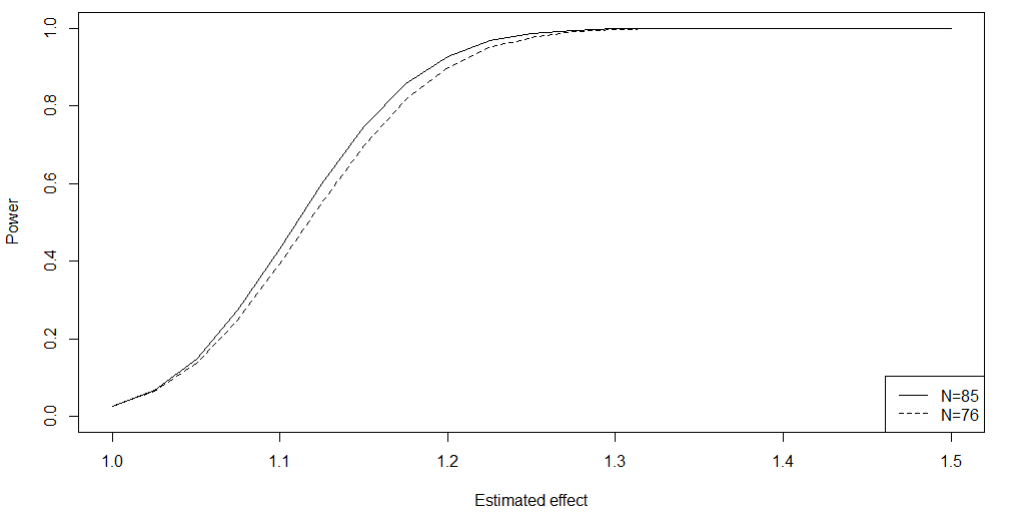
Power for estimated effect sizes for analyses of whole blood samples including either 85 or 76 participants.

The genome-wide transcriptional response to HBO_2_ has not previously been addressed in any type of tissue or disease. Hence, the number of biological replicates necessary to observe a significant difference in gene expression before and after HBO_2_ treatment is unknown. Based on the depth of gene coverage for all expressed genes and the overall coefficient of variation for the blood samples found in the pilot study, we estimated the sample size for the 2 conditions according to published methods for the primary outcome of this paper’s “study 1” [40]. Assuming a coefficient of variation of 0.34 for the whole-blood samples, a depth of coverage of human reads of 218, a risk of type I error of 5%, and a risk of type II error of 20% (with a power of 80%), the number of participants per group required to detect a 25% difference between conditions is 38, giving a total sample size of 76 participants. However, the differential expression analysis will focus on the majority of genes that are better behaved, and we therefore expect a stronger power [40]. On the other hand, we also expect that approximately 10% of samples will fail quality control. Therefore, we decided to include 85 participants in the study. Figure 3 depicts the power for estimated effect sizes. Regardless of the estimated effect sizes, all biological and clinically relevant findings will be reported in the exploratory analysis.

## Results

Patient recruitment in the clinical setting was completed in 2017. Screening of the resulting biobank for eligible participants for the HBOmic study according to the inclusion and exclusion criteria has been completed. Informed consent for participation in the HBOmic study has been obtained from all eligible study participants, and the first participant was enrolled on July 27, 2021. Data analysis is expected to begin during autumn 2022, with publication of results immediately thereafter.

## Discussion

In the HBOmic study, we anticipate being able to identify NSTI endotypes with differential responses to HBO_2_ treatment based on transcriptional responses. We will analyze longitudinal genome-wide transcriptional data in systematically collected daily blood and infected-tissue samples from patients with NSTI, which will allow us to capture immunomodulatory changes associated with critical events following HBO_2_ treatment in this rare disease complex. NSTI is a heterogeneous and dynamic disease, and the biological variability of the specimens under study is expected to be high. To account for this, we have included a relatively large cohort of septic patients with NSTI. The study is highly feasible, because we have already performed the described data validation, including testing the quality of the samples and the resulting sequencing data. In the pilot study, 1 sample failed quality control and was dismissed from further analysis. We have therefore estimated that 10% of samples may fail quality control, which we have accounted for in the sample size estimation. We will identify molecular differences with a broad-spectrum analysis of transcriptomic data with a systems approach, in which specific parameters have not been chosen as they will be by default everything or anything. In that sense, this data-driven research will deliver unbiased and unprecedented information about immunomodulatory changeability during disease progression and treatment. On the other hand, this approach has inherent limitations and biases, including gene panel selection bias and sequencing bias in library construction and amplification bias. To minimize technical biases during the processing of this high number of samples, we will carefully design our batches with bridging and perform sensitivity analyses in response to degradation markers. A systems approach to this heterogeneous disease complex implies the investigation of many markers, which might make it challenging to identify the most relevant biomarkers and increases the risk of nonreproducible results. Future approaches could include identifying genes predictive of group membership in our cohort, and then, in a validation cohort, assigning group membership to individuals based on their expression of the predictive gene set and evaluating the robustness of the prediction. Another future approach could be to perform multiomic analyses. Combining transcriptomics with genomics could shed light on the link between genotype and any phenotypes identified in the clustering analysis. For this, mapping to expression quantitative trait loci would allow us to focus on genes that are expressed differently in blood and infected tissue. This would allow distinguishing responders and nonresponders on the genetic level. On the other end of the omics sequence, proteomics would give insight into protein modifications, and in combination with our differential expression analysis proteomics would give a more precise view of the differential protein abundance and thereby strengthen the identification of candidate biomarkers for clinical trials. However, transcriptomics is the first step in which environmental effects can impact the translation from DNA to cellular function, and it thereby constitutes an appropriate initial application.

The availability of transcriptomic data from pathogens and hosts from 2 different tissues (ie, whole blood and soft tissue) at separate time points with an intermediate intervention is unique and will provide real-time snapshots of cellular and extracellular signaling pathways that are up- and downregulated in different clinical subgroups. The HBOmic study will provide new insights into personalized patient management in NSTIs and selection for future clinical trials.

## Abbreviations

cDNA: copy DNA
HBO_2_: hyperbaric oxygen
HBOmic: Effects of Hyperbaric Oxygen Treatment Studied With Omics
INFECT: Systems Medicine to Study Necrotizing Soft Tissue Infections
mRNA: messenger RNA
NSTI: necrotizing soft tissue infection
RQN: RNA quality number
rRNA: ribosomal RNA
TIN: transcript integrity number

## Data Availability

The human sequencing data underlying the results of this study will not be openly available due to their sensitive nature. The Danish National Committee on Health Ethics has rejected making the human genetic data generated in this study publicly available in any form. Bacterial sequence data that support the findings of the studies described in this protocol will be deposited in a public repository (such as ArrayExpress or GEOCIBEX).
